# Virus-like particles derived from *Pichia pastoris*-expressed dengue virus type 1 glycoprotein elicit homotypic virus-neutralizing envelope domain III-directed antibodies

**DOI:** 10.1186/s12896-016-0280-y

**Published:** 2016-06-14

**Authors:** Ankur Poddar, Viswanathan Ramasamy, Rahul Shukla, Ravi Kant Rajpoot, Upasana Arora, Swatantra K. Jain, Sathyamangalam Swaminathan, Navin Khanna

**Affiliations:** Recombinant Gene Products Group, International Centre for Genetic Engineering & Biotechnology, Aruna Asaf Ali Marg, New Delhi, 110067 India; Department of Biotechnology, Jamia Hamdard, Hamdard Nagar, New Delhi, 110062 India; Department of Biochemistry, HIMSR, Jamia Hamdard, New Delhi, 110062 India; Department of Biological Sciences, Birla Institute of Technology & Science, Jawahar Nagar, Shamirpet, Hyderabad, 500078 India

**Keywords:** Virus-like particles, Dengue virus, Envelope glycoprotein, Envelope domain III, *Pichia pastoris*, Neutralizing antibody, Antibody dependent enhancement

## Abstract

**Background:**

Four antigenically distinct serotypes (1–4) of dengue viruses (DENVs) cause dengue disease. Antibodies to any one DENV serotype have the potential to predispose an individual to more severe disease upon infection with a different DENV serotype. A dengue vaccine must elicit homotypic neutralizing antibodies to all four DENV serotypes to avoid the risk of such antibody-dependent enhancement in the vaccine recipient. This is a formidable challenge as evident from the lack of protective efficacy against DENV-2 by a tetravalent live attenuated dengue vaccine that has completed phase III trials recently. These trial data underscore the need to explore non-replicating subunit vaccine alternatives. Recently, using the methylotrophic yeast *Pichia pastoris*, we showed that DENV-2 and DENV-3 envelope (E) glycoproteins, expressed in absence of prM, implicated in causing severe dengue disease, self-assemble into virus-like particles (VLPs), which elicit predominantly virus-neutralizing antibodies and confer significant protection against lethal DENV challenge in an animal model. The current study extends this work to a third DENV serotype.

**Results:**

We cloned and expressed DENV-1 E antigen in *P. pastoris*, and purified it to near homogeneity. Recombinant DENV-1 E underwent post-translational processing, namely, signal peptide cleavage and glycosylation. Purified DENV-1 E self-assembled into stable VLPs, based on electron microscopy and dynamic light scattering analysis. Epitope mapping with monoclonal antibodies revealed that the VLPs retained the overall antigenic integrity of the virion particles despite the absence of prM. Subtle changes accompanied the efficient display of E domain III (EDIII), which contains type-specific neutralizing epitopes. These VLPs were immunogenic, eliciting predominantly homotypic EDIII-directed DENV-1-specific neutralizing antibodies.

**Conclusions:**

This work demonstrates the inherent potential of *P. pastoris*-expressed DENV-1 E glycoprotein to self-assemble into VLPs eliciting predominantly homotypic neutralizing antibodies. This work justifies an investigation of the last remaining serotype, namely, DENV-4, to assess if it also shares the desirable vaccine potential manifested by the remaining three DENV serotypes. Such efforts could make it possible to envisage the development of a tetravalent dengue vaccine based on VLPs of *P. pastoris*-expressed E glycoproteins of the four DENV serotypes.

**Electronic supplementary material:**

The online version of this article (doi:10.1186/s12896-016-0280-y) contains supplementary material, which is available to authorized users.

## Background

Dengue disease is a global public health threat caused by four distinct serotypes of dengue viruses (DENV-1, −2, −3 and −4) spread primarily by *Aedes aegypti* mosquito [1]. According to a report in the year 2013, the number of annual global dengue infections was estimated to be ~400 million, with ~96 million clinically apparent infections [2]. DENV is a positive sense RNA virus with ~11 kilo base (kb) genome, which encodes three structural and seven non-structural proteins [3]. Dengue disease can vary from mild dengue fever to severe, life-threatening syndromes, dengue hemorrhagic fever and dengue shock syndrome [1, 4, 5]. DENV infection, which results in lifelong homotypic immunity, affords only transient heterotypic immunity [6]. In fact, heterotypic antibodies are implicated in promoting DENV uptake during a secondary infection with a different serotype, through Fc receptor pathway and contributing to increased viral load, leading to more severe disease [1, 7]. To preclude the possibility of such antibody-mediated enhancement (ADE) of dengue disease, it is believed that a safe dengue vaccine must be ‘tetravalent’, affording simultaneous type-specific (homotypic) protection against each of the four DENV serotypes. This requirement poses a major hurdle to dengue vaccine development [8]. Many live attenuated vaccine candidates are in clinical development. Of these, Sanofi’s Chimeric Yellow Fever Dengue-Tetravalent Dengue Vaccine (CYD-TDV) has recently completed Phase III trials [9, 10] and is currently being introduced in some dengue-endemic countries [11]. However, this vaccine candidate has certain limitations. It needs to be administered in 3 doses over a one year period. CYD-TDV is not as effective in dengue-naïve individuals, as in those with a history of prior dengue exposure. Its efficacy against DENV-2 is very low, despite its apparent capacity to induce serotype 2-specific neutralizing antibodies [9, 10]. Efforts to understand this, using a mouse model of ADE, strongly suggest that while homotypic neutralizing antibodies do not cause ADE, heterotypic neutralizing antibodies do, at certain concentrations [12]. This underscores the requirement for a dengue vaccine to elicit homotypic neutralizing antibodies to each of the four prevalent DENV serotypes to be both safe and efficacious.

Using a non-replicating subunit vaccine approach, we showed recently that it is possible to elicit predominantly homotypic neutralizing antibody titers using *P. pastoris*-expressed envelope (E) glycoproteins of DENV-2 and DENV-3 in mice [13, 14]. The DENV-E protein is the major surface exposed glycoprotein of about ~500 amino acid (aa) residues, which is organized into three discrete domains, EDI, EDII and EDIII [15]. Of these, EDIII is critical from a vaccine perspective, as it is not only involved in host receptor recognition and virus entry into susceptible cells, but also in the induction of potent type-specific virus neutralizing antibodies [16, 17]. Interestingly, our work showed that the N-terminal 80 % of the E glycoprotein (ectodomain) of DENV-2 [13] and DENV-3 [14], which encompasses EDIII, produced using *P. pastoris* assembled into discrete virus-like particles (VLPs). These VLPs served as efficient display platforms for EDIII and elicited potent, homotypic virus-neutralizing antibodies [13, 14], capable of conferring significant protection against live virus challenge in an animal model [13]. Unlike dengue virion particles which contain E along with another structural protein, prM, implicated in the induction of ADE-mediating antibodies [18, 19], these VLPs contain only the E glycoprotein. Thus, the *P. pastoris*-derived DENV E VLPs, lacking the ADE-associated prM protein, offer a significant safety advantage. This has provided us the rationale to explore the feasibility of developing DENV E VLPs corresponding to the remaining two serotypes as well, so that we may eventually develop a tetravalent DENV E VLP-based vaccine candidate.

In this paper, we specifically focus on expressing and characterizing DENV-1 E glycoprotein using *P. pastoris* to address the following specific questions: (i) Will DENV-1 E glycoprotein, expressed in *P. pastoris*, also possess VLP-forming potential? (ii) Would these VLPs preserve the antigenic integrity of the critical virus-neutralizing epitopes? (iii) Would the VLPs be immunogenic, and if so, would the antibodies elicited be homotypic? The work described in this paper demonstrates the feasibility of creating *P. pastoris*-expressed DENV-1 E-based VLPs that are capable of eliciting EDIII-directed type-specific neutralizing antibodies and moves a step closer to developing a tetravalent dengue VLP vaccine candidate.

## Results

### Recombinant DENV-1 E undergoes proper processing and glycosylation in *P. pastoris* and self- assembles into stable VLPs

**Fig. 1:**
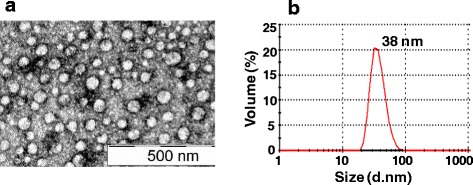
Investigation of VLP formation by purified DENV-1 E protein. **a** EM analysis of DENV-1 E VLPs by negative staining with 1 % uranyl acetate. **b** DLS analysis of particle size distribution by volume

**Fig. 2:**
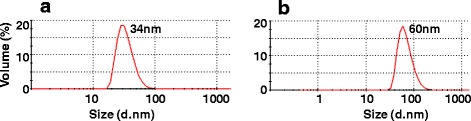
VLPs stability study. DLS analysis of DENV-1 E VLP size distribution by volume after incubating them for 2 weeks at (**a**) 4 °C or (**b**) 37 °C

**Fig. 3:**
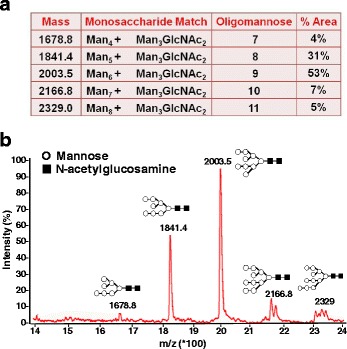
MALDI-TOF-MS analysis of N-linked Oligosaccharides of *P. pastoris*-expressed DENV-1 E protein. **a** Tabular representation of molecular weights of sugars attached to DENV-1 E protein with their respective structures, number of mannose residues and their percent area (% area). The % area corresponds to the relative proportion of the oligosaccharide out of total oligosaccharide populations. **b** Graphical representation of peaks corresponding to different molecular weight sugars with their respective intensities on y-axis and corresponding mass/charge (m/z) ratios on x-axis

A synthetic *DENV-1 E* gene encoding the C-terminal 34 aa residues of prM as a signal peptide, followed by the N-terminal 395 aa ectodomain of the E glycoprotein of DENV-1, pentaglycyl linker and a stretch of 6 histidine residues was cloned into *pPICZA* vector and expressed in KM71H strain of *P. pastoris* (Additional file 1: Figure S1). The expressed recombinant DENV-1 E protein which was associated with the membrane fractions was affinity-purified under denaturing conditions (Additional file 1: Figure S2). As observed earlier for the E proteins of DENV-2 [13] and DENV-3 [14], the DENV-1 E protein was also processed properly by *P. pastoris*, based on N-terminal sequence analysis, which showed that the prM-derived signal peptide had been removed. Electron microscopic (EM) and dynamic light scattering (DLS) analyses revealed the purified DENV-1 E protein to be assembled into ~38 nm VLPs (Fig. 1). This, taken together with our previous observations, confirms the inherent ability of *P. pastoris*-DENV E ectodomain to self-assemble into VLPs in the absence of prM protein. Further, we observed that the VLP nature was preserved upon storage at 37 °C for 14 days (Fig. 2). In fact, DLS analysis of stored VLPs showed them to be comparatively larger, suggesting that these VLPs mature further, as observed earlier for other viral antigen-derived VLPs [13, 20]. N-linked oligosaccharide profiling by MALDI-TOF mass spectroscopy revealed the presence of mannosylated population of glycans linked to DENV-1 E protein. These glycans consisted of a common pentasaccharide core (Man_3_GlcNAc_2_) with 4–8 additional mannose residues (Fig. 3). The precise significance of such *P. pastoris*-mediated glycosylation of the E glycoprotein, from a vaccine perspective, is not clear. Presumably, it may have a role in antigen uptake and processing [21].

### DENV-1 E VLPs display key viral epitopes on their surface

The DENV-1 E VLPs were antigenically characterized with a panel of murine and human mAbs [17, 22–28] specific to various surface exposed epitopes of the virus (Table 1). Of these mAbs, those that bind to the lateral ridge (LR) of EDIII are notable. The EDIII LR epitope, mapping to aa residues 301, 302, 329, 330 and 386, elicits potent virus-neutralizing antibodies [16, 17]. Using an indirect ELISA format described recently [14], we found that the DENV-1 E VLPs reacted strongly with DENV-1-specific mAb E103. This mAb which binds to aa residues on BC and DE loop of EDIII LR epitope, is documented to neutralize multiple DENV-1 genotypes, namely, TVP-2130, 16007, TVP-5175, West Pac-74 and 3146- SL [17]. Consistent with its type-specificity, mAb E103 does not recognize DENV-2 E and DENV-3 E VLPs. This demonstrates that the DENV-1 E VLPs retain the type-specific EDIII-LR epitope of DENV-1 and display it efficiently to mAb E103. The EDIII-LR epitope displayed on the DENV-1 E VLP surface largely retains its serotype 1-specific antigenic character is corroborated by the lack of significant ELISA reactivity towards DENV-2 mAb 3H5 [22], DENV-3 mAb 8A1 [23] and DENV-4 mAb E88 [24]. Apart from the LR epitope, the VLPs also displayed other EDIII-specific epitopes efficiently, based on the ELISA reactivity, manifested towards DENV-1 mAbs E24, E29 and E37 [17]. Consistent with these observations several cross-reactive murine and human mAbs specific to EDIII, within (mAb E77) and outside the LR (mAbs 12C1, h-2 J20), also recognized the DENV-1 E VLPs efficiently. Additionally, the VLPs also displayed EDI/II epitopes, indicated by their reactivity towards mAb E17 [25], and fusion-loop epitopes, based on their recognition by mAbs h-1 M7 [27] and 4G2 [22]. Consistent with the design of DENV-1 E antigen, we did not detect any ELISA reactivity of the DENV-1 E VLPs towards prM-specific mAb 2 K2 [28]. Collectively, these data show that the DENV-1 E VLPs largely retain the epitope architecture of the virion. Notably, they display EDIII and its type-specific epitopes quite efficiently on their surface.

**Table 1:** Analysis of antigenic integrity of DENV-1 E VLPs^*a*^

Type-specific murine mAbs					
mAb^*b*^	Epitope Specificity	Absorbance, 450 nm			
		DENV-3 E	DENV-2 E	DENV-1E	HBsAg
DENV1 E24	EDIII	0.03	0.02	1.23	0.03
DENV1 E29	EDIII	0.09	0.03	1.87	0.03
DENV1 E37	EDIII	0.03	0.02	0.71	0.04
DENV1 E103	EDIII, LR	0.06	0.06	3.48	0.02
DENV-2 3H5	EDIII, LR	0.06	3.46	0.14	0.06
DENV-3 8A1	EDIII, LR	1.35	0.02	0.08	0.03
DENV-4 E88	EDIII, LR	0.03	0.07	0.05	0.05
Cross-reactive murine & human mAbs					
mAb	Epitope Specificity	Absorbance, 450 nm			
		DENV-3 E	DENV-2 E	DENV-1E	HBsAg
E17^*c*^	EDI/II	3.45	3.47	3.42	0.07
4G2^*d*^	Fusion loop	0.83	0.20	0.23	0.06
12C1^*d*^	EDIII, not LR	3.72	3.86	3.76	0.02
E77^*Com*^	EDIII, AS, LR	2.41	1.21	3.50	0.04
h-1M7^*d*^	Fusion loop	2.48	3.03	2.97	0.01
h-2 J20	EDIII	3.58	3.46	3.67	0.06
h-2 K2	prM	0.04	0.03	0.03	0.06

^a^Determined by indirect ELISA using *P. pastoris*-produced purified DENV E VLPs as coating antigens. For comparison, corresponding data for DENV-2 E and DENV-3 E VLPs [14] are shown

^*b*^The type-specific mAbs used are described in literature: DENV-1 mAbs E24, E29, E37 & E103 [17]; DENV-2 mAb 3H5 [22]; DENV-3 mAb 8A1 [23]; DENV-4 mAb E88 [24] . Cross-reactive mAbs: E17 [25]; 4G2 [22]; 12C1 [23]; h-1 M7 [27]; h-2 J20 [26] & h-2 K2 [28]; All mAb are murine, except the last 3 which are human (prefixed ‘h’)

^*c*^Sub-complex-specific mAbs; E17 recognizes EDI/II of DENV-1 and DENV-3

^*d*^Complex-specific mAbs; 4G2 and h-1 M7 bind the fusion loop of all four DENV Es; 12C1 binds recombinant EDIII (outside the LR epitope) of all four DENV serotypes

### DENV-1 E VLPs elicit EDIII-directed type-specific virus-neutralizing antibodies in mice

**Fig. 4:**
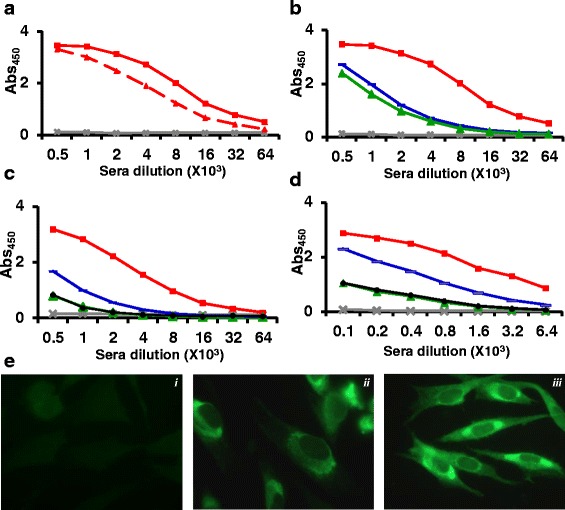
Characterization of antibodies generated by DENV-1 E VLPs. Pooled sera from mice immunized with DENV-1 E VLPs, collected on day 37 (*dashed line*) and day 100 (*solid line*) were analysed in indirect ELISA format using DENV-1 E VLPs as the coating antigen (**a**). Pooled day 100 immune serum was evaluated for reactivity using DENV-E VLPs (**b**), recombinant EDIIIs (**c**), and intact DENVs (**d**) taken as coating antigens, corresponding to serotypes 1 (*red curve*), 2 (*green curve*), 3 (*blue curve*) and 4 (*black curve*). Grey lines (in all panels) represented serum from PBS-immunized BALB/c mice. **e** Indirect immunofluorescence analysis of DENV-1 infected BHK-21 cells using (i) PBS-immunized serum (ii) 4G2 mAb (iii) anti-DENV-1 E VLP antiserum taken as the source of primary antibodies. Anti-mouse IgG-FITC conjugate was used to visualize bound antibodies

**Fig. 5:**
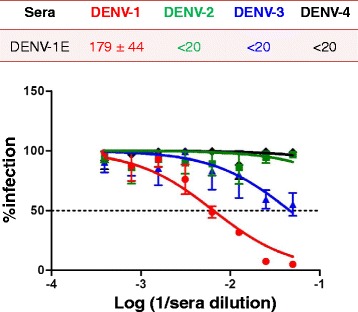
Determination of virus neutralization potency of antibodies induced by DENV-1 E VLPs. Immune sera were serially diluted two-fold and pre-incubated separately with DENV-1 (*red curve*), DENV-2 (*green curve*), DENV-3 (*blue curve*) and DENV-4 (*black curve*). The antibody-virus complexes were allowed to infect Vero cells and neutralizing antibody titers (FNT_50_) were determined by using FACS analysis. The Y and X axes of the graph represent % infected cells and log of reciprocal of sera dilution, respectively. The dotted horizontal line in the graph represents 50 % infection. The table on top of the panel lists the FNT50 titers of DENV-1 E antiserum against all the four serotypes

**Fig. 6:**
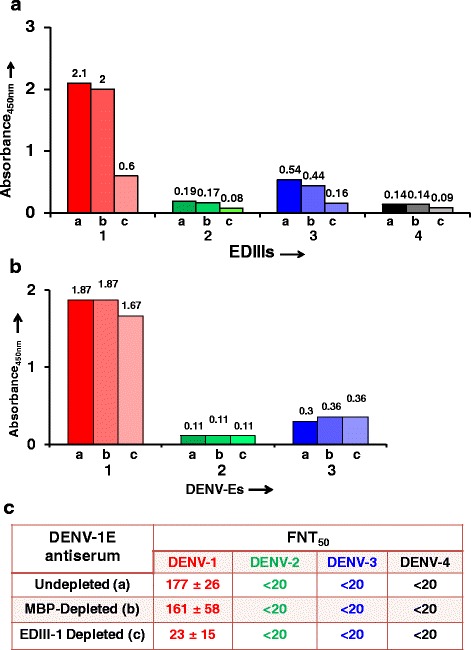
Analysis of the role of anti-EDIII antibodies in virus neutralization: DENV-1 E VLP antiserum was used without prior depletion (*a*) or treated as follows. It was incubated with immobilized MBP (*b*) or immobilized MBP-EDIII-1 (*c*) matrix. Residual antibody titers in these three DENV-1 E VLP antiserum treatment groups (*a*, *b* and *c*), were analyzed in indirect ELISA using purified recombinant MBP-EDIII-1, −2, −3, −4 protein (panel **a**) or DENV-1, −2, −3 E VLPs (panel **b**). Residual neutralizing antibody titers (FNT50) in the same three sera groups were estimated using the FACS assay (Panel **c**)

The observation that DENV-1 E VLPs retain many epitopes recognized by neutralizing mAbs suggested that they must be immunogenic and be capable of anti-DENV-1 immune response. To ascertain this, BALB/c mice were immunized with DENV-1 E VLPs on days 0, 30 and 90, as reported earlier [13, 14]. Mice were bled on days 37 and 100 and sera were evaluated for presence of antibodies by indirect ELISA using purified DENV-1 E as the coating antigen (Fig. 4). The ELISA reactivity of day 100 serum on DENV-1 E VLPs was significantly higher than day 37 serum (Fig. 4a), indicating that day 90 dose boosted the immune response. Next, we evaluated the extent of cross-reactivity of anti-DENV-1 E antiserum towards DENV-2 E [13] and DENV-3 E [14] VLPs described earlier. We observed that antibodies elicited by DENV-1 E do recognize and bind to DENV-2 E and DENV-3 E (Fig. 4b). However, the heterotypic ELISA reactivity was noticeably lower than the homotypic ELISA reactivity. The heterotypic ELISA reactivity is presumably due to the presence of cross-reactive (EDI/II and FL) epitopes on the VLPs observed in this study (Table 1). This pattern of cross-reactivity was mirrored in indirect ELISAs performed using recombinant EDIII proteins of the four serotypes (Fig. 4c) or the intact viruses themselves (Fig. 4d) as the coating antigens. The observation that anti-DENV-1 E VLP-antibodies recognize DENV-1 (Fig. 4d) was corroborated by IFA, as depicted in Fig. 4e. This experiment showed that antibodies elicited by DENV-1 E VLPs recognized and bound to DENV-1 in infected BHK-21 cells efficiently (Fig. 4e, panel iii). Next, we assessed the virus-neutralizing efficacy of these DENV-1 E VLP-induced antibodies using a FACS based neutralization assay [13, 14]. Testing each one of the four DENVs in this assay, we found that the anti-DENV-1 E VLP antiserum possessed FNT_50_ titre of 179 against DENV-1, with no discernible neutralizing titers towards any of the remaining three DENV serotypes (Fig. 5). This observation of homotypic neutralizing activity is essentially consistent with our observations with DENV-2 E and DENV-3 E VLPs reported earlier [13, 14]. As these studies had revealed that the homotypic neutralizing antibody titers are predominantly EDIII-focused, we sought to ascertain this in case of DENV-1 E-induced antibodies as well. Thus, as done earlier (14), a depletion assay was performed wherein DENV-1 EDIII-specific antibodies were removed from anti-DENV-1 E VLP immune serum by incubating it with a maltose-binding protein (MBP) fusion of DENV-1-derived recombinant EDIII immobilized on amylose resin. As expected, following this depletion the anti-DENV-1 E VLP antiserum manifested very little reactivity in an ELISA using EDIII as the coating antigen (Fig. 6a). The question we addressed at this point was, what is the effect of depleting anti-EDIII antibodies on ELISA reactivity towards the E glycoprotein? A similar ELISA, performed using recombinant DENV E VLPs as the capture antigen revealed that EDIII antibody depletion did not affect DENV-1 E specific antibody titers (Fig. 6b). Having verified that the anti-DENV-1 E antiserum had been specifically depleted of EDIII-directed antibodies alone, we next determined residual virus-neutralizing antibody titers in it (Fig. 6c). This experiment revealed significant reduction in DENV-1 virus-specific FNT_50_ titer, suggesting that majority of DENV-1 E neutralizing immune response is mediated by EDIII-directed antibodies.

## Discussion

Dengue has recently been recognized as one of the fastest spreading vector-borne diseases [29]. Since, a preventive dengue vaccine has been an unmet need for very long has spurred the introduction of a live attenuated vaccine (CYD-TDV) recently in a couple of dengue-endemic countries, despite sub-optimal efficacy in phase III clinical trials [9, 10]. Attempts to understand the basis of lack of vaccine efficacy, have revealed that neutralizing antibody titers elicited by CYD-TDV are directed predominantly towards one serotype [30], presumably stemming from viral interference [31–33]. This taken in the context of another recent study, which demonstrated that neutralizing antibodies need to be homotypic to preclude the possibility of ADE [12], strongly suggests that it is important for a safe and effective dengue vaccine to elicit type-specific neutralizing antibodies to each of the four prevalent DENV serotypes. This situation warrants a continued search for alternate non-replicating vaccine candidates that may hopefully eliminate interference and the associated potential safety concerns [34].

Our recent work has shown that in the context of dengue, VLPs offer such an alternate option. These VLPs are spherical, non-replicative, lack viral genomic RNA, and are highly immunogenic. We reported earlier that the E glycoproteins of DENV-2 and DENV-3, when expressed in *P. pastoris*, in the absence of the companion structural protein, prM, assemble into discrete VLPs [13, 14]. Several attributes of these VLPs are noteworthy from the perspective of a dengue vaccine candidate. First is the observation that prM, a minor structural protein necessary for virion maturation which is documented to elicit antibodies implicated in ADE [18, 19], is not required for VLP formation. Second, these VLPs were shown to preserve the neutralizing epitopes of the cognate DENV serotype. Interestingly, these VLPs displayed EDIII efficiently. This is significant as EDIII is implicated in host receptor recognition and contains multiple type-specific neutralizing epitopes [16, 17]. Third, though these VLPs elicited antibodies against all the DENV serotypes, they neutralized only DENV-1, mediated by anti-EDIII antibodies. This is of significance as only cross-reactive virus-neutralizing antibodies appear to be associated with ADE [12]. Fourth, in an animal challenge model, DENV-2 E VLPs afforded statistically significant protection. Collectively, these findings underlie our efforts to develop a tetravalent VLP vaccine candidate based on *P. pastoris*-expressed DENV E glycoproteins. Towards this objective, we have extended our VLP vaccine work to a third DENV serotype, namely, DENV-1, in this paper.

The design of the DENV-1 E antigen gene was exactly similar to that of DENV-2 and DENV-3 E antigens described previously [13, 14]. Moreover, a multiple sequence alignment of the four DENV-E aa sequences (of the specific genotypes of the four serotypes) revealed 60–80 % similarity (Additional file 1: Figure S3), which is in compliance with the reports in literature [35]. The DENV-1 E antigen was provided with an N-terminal DENV-1 prM-derived signal peptide and a C-terminal 6x His tag and expressed in *P. pastoris* by methanol induction. As reported in the earlier studies, the DENV-1 E protein was processed similarly by *P. pastoris*, in that the prM peptide was cleaved off and the mature protein glycosylated. Consistent with the behavior of DENV-2 E and DENV-3 E proteins, the DENV-1 E protein also self-assembled into VLPs, during downstream processing, as evidenced by EM and DLS analyses. These VLPs were stable and appeared to mature to a greater degree of size homogeneity during storage. Probing the surface of these VLPs using several different type-specific and cross-reactive human and murine mAbs demonstrated that several of the epitopes that exist on the DENV-1 virion surface are present on the DENV-1 E VLPs as well. Of note was the observation that EDIII was displayed on the VLP surface with its LR epitope, critical for the induction of type-specific neutralizing antibodies, intact and freely accessible. Consistent with this, we found that the DENV-1 E VLPs elicited homotypic neutralizing antibodies. EDIII antibodies elicited by these VLPs were almost exclusively responsible for neutralizing DENV-1, as evident from abrogation of neutralizing antibody titer in immune sera following antibody depletion using immobilized DENV-1 EDIII antigen. Like the antibodies induced by DENV-3 E [14], antibodies induced by DENV-1 E VLPs also did not cause heterotypic enhancement of DENV infection at 1:20 sera dilution (data not shown). This underscores the utility of eliciting serotype-specific neutralizing antibodies to preclude heterotypic ADE. The data thus far on *P. pastoris*-produced DENV-1 E VLPs essentially mirror our earlier findings with DENV-2 and DENV-3 E VLPs and provide the rationale for extending this work to the E glycoprotein of the last remaining serotype, DENV-4.

## Conclusion

We have evaluated the potential of *P. pastoris*-expressed DENV-1 E glycoprotein-based VLPs as a potential vaccine candidate. We show that these DENV-1 E VLPs generate predominantly EDIII-directed, DENV-1 serotype specific, neutralizing immune antibody responses. The complete absence of prM would be an in-built safety advantage. Multimeric presentation of antigenic epitopes with predominance of EDIII determinants on DENV-1 E VLPs may overcome the problem of low immunogenicity associated with monomeric subunit based vaccine candidates. Stability of VLPs at higher temperature would help in reducing the challenges faced during vaccine storage and administration. The *P. pastoris*-expressed E glycoproteins of three DENV serotypes so far studied manifest several attributes, desirable from a vaccine standpoint, namely, the capacity to: (i) form immunogenic VLPs, (ii) efficiently display EDIII that contains type-specific neutralizing epitopes, and (iii) induce homotypic neutralizing antibody titers. This, coupled to the high expression potential of the *P. pastoris* host system strongly suggests that extension of this work to the last remaining serotype, DENV-4, may set the stage for developing a safe, effective and inexpensive VLP dengue vaccine candidate.

## Methods

### *DENV-1 E* gene, expression plasmid, cell line, virus and other reagents

*DENV-1 E* gene (~1.3 kb: Genbank accession no: JX292264) codon-optimized for expression in *P. pastoris* system was custom synthesized by GeneScript, New Jersey, USA. *E.coli* strain DH5α, *P. pastoris* strain KM71H and expression plasmid *pPICZA* were procured from Invitrogen Life Technologies, Carlsbad, USA. Vero and BHK-21 cell lines were purchased from American Type Cell Culture (ATCC), Virginia, USA. WHO reference viral strains DENV-1 (WP 74), DENV-2 (S16803), DENV-3 (CH53489), DENV-4 (TVP-360); *E. coli* clones expressing MBP (Maltose Binding Protein) and MBP-EDIII-1 (EDIII domain of DENV-1 fused with MBP) fusion proteins were received from Dr. Aravinda de Silva, University of North Carolina (UNC), USA. All mAbs used in this study were the same as before [13, 14]. Concanavalin A (Con A) peroxidase conjugate was purchased from Sigma-Aldrich. Goat anti-mouse and anti-human IgG monoclonal antibody-HRPO conjugates and anti-mouse fluorescein isothiocyanate (FITC) conjugate were procured from Life Technologies, USA and Merck, Germany, respectively. Ni-NTA resin was procured from Qiagen, Hilden, Germany. High binding, polystyrene ELISA plates were purchased from Corning Incorporated, USA. N-linked oligosaccharide profiling was performed at GlycoSolutions Corp., Marlborough, USA.

### *DENV-1 E* gene cloning, expression, purification and characterization

*DENV-1 E* gene was cloned at *EcoRI* and *NotI* site of *pPICZA* expression plasmid. The resultant plasmid was linearized with *Sac I*, electroporated into *P. pastoris* strain KM71H and expressed under the control of *AOX1* promoter as done previously for DENV-2 E [13] and DENV-3 E [14]. Transformants were selected on zeocin plates and induced with 1.5 % methanol every 24 h for 72 h. DENV-1 E was purified from induced *P. pastoris* cells using Ni-NTA chromatography under denaturing conditions as described previously [13]. Purified protein was analyzed on Coomassie gel and Western blot using anti-EDIII mAbs. The identity and similarities between the amino acid sequences of DENV-1 (West Pac-74), DENV-2 (New Guinea C), DENV-3 (H87) and DENV-4 (Dominica) derived recombinant E genes with GenBank accession number JX292264, JX292265, JX292266, JX292267 respectively was determined by multiple sequence alignment using Clustal W tool. Further, DENV-1 E protein was evaluated for assembly into VLPs by EM and DLS studies [14]. Briefly, EM studies were performed by coating the purified and dialyzed DENV-1 E (at 5–10 ug/ml) on carbon-formvar grid, followed by negative staining with 1 % uranyl acetate, which were examined under electron microscope. Malvern Zetasizer NanoZ was used to assess the particle size and distribution of purified and dialysed DENV-1 E VLPs by dynamic light scattering. The stability of VLPs after incubation at 37 °C for 14 days was also evaluated by DLS studies as described before [14]. Further, the protein was characterized by MALDI-TOF-Mass spectroscopy to determine the structure of N-linked glycan attached to protein. The integrity of conformational epitopes of DENV-1 E protein was evaluated using a panel of murine and human mAbs by indirect ELISA as reported previously [14]. Further, the reactivity of antibodies in the DENV-1 E VLP immunized mice sera was evaluated by indirect ELISA and IFA as reported previously [13, 14]. BALB/c mice (*n* = 6) were immunized with 20 μg of DENV-1 E VLP on day 0 and subsequently boosted on days 30 and 90. Mice were bled on days 37 and 100 for seroanalysis. The neutralization efficacy of DENV-1 E sera was evaluated against all the four DENV serotypes on Vero cell line by FACS based neutralization assay as described before [13, 14]. Further, the proportion of EDIII-directed neutralizing antibody titers in DENV-1 E serum was evaluated by pre-incubating it with amylose resin coated with MBP-fused in-frame to DENV-1 EDIII, prior to use in FACS based DENV neutralization assay [14]. FACS data were analysed using FlowJo software.

## Abbreviations

ADE, Antibody dependent Enhancement; AOX, Alcohol oxidase; CYD-TDV, Chimeric yellow fever dengue-tetravalent dengue vaccine; DENV, Dengue virus; DLS, Dynamic light scattering; E, Envelope; ED, Envelope domain; EM, Electron microscopy; FNT, Flow cytometry based neutralization titer; IFA, Immunofluorescence assay; LAV, Live attenuated vaccine; mAb, monoclonal antibody; MBP, Maltose binding protein; prM, pre-membrane; VLP, Virus like Particle

## Additional file

Additional file 1: Figure S1. Cloning and expression of *DENV-1 E* gene into shuttle vector *pPICZA.* **Figure S2**. Purification and characterization of recombinant DENV-1 E antigen. **Figure S3**. Sequence alignment of *P.* *pastoris* optimized DENV-1, 2, 3, 4 E showing similarities and differences in the amino acid sequences between four dengue serotypes. (DOC 1608 kb)
