# Supplementary material for: Virus-like particles derived from Pichia pastoris-expressed dengue virus type 1 glycoprotein elicit homotypic virus-neutralizing envelope domain III-directed antibodies
Source: BMC Biotechnol. 2016 Jun 14;16:50. doi: 10.1186/s12896-016-0280-y (PMC4908714; doi:10.1186/s12896-016-0280-y)
Supplement: Additional file 1: Figure S1. — Cloning and expression of DENV-1 E gene into shuttle vector pPICZA. Figure S2. Purification and characterization of recombinant DENV-1 E antigen. Figure S3. Sequence alignment of P. pastoris optimized DENV-1, 2, 3, 4 E showing similarities and differences in the amino acid sequences between four dengue serotypes. (DOC 1608 kb) [file 12896_2016_280_MOESM1_ESM.doc]

**Additional File 1**

**Virus-like particles derived from *Pichia pastoris*-expressed dengue virus type 1 glycoprotein elicit homotypic virus-neutralizing envelope domain III-directed antibodies**

Poddar et al.

**Figure S1: Cloning and expression of *DENV-1 E* gene into shuttle vector *pPICZA*** (A) Custom-synthesized *DENV-1 E* gene was cloned in shuttle vector *pPICZA* at *EcoRI* and *NotI* sites between the *AOX1* promoter (5’ AOX1) and the *AOX1* transcriptional terminator (TT). Zeocin and pUC *Ori* denote the zeocin selection marker and the plasmid origin of replication, respectively. *DENV-1 E* gene comprised of nucleotide sequences encoding 34 aa residues of C-terminus of prM (blue box), 395 aa of E ectodomain (red box), pentaglycine linker (orange) and a stretch of six histidine aa residues (green). (B) Predicted amino acid sequence encoded by custom synthesized *DENV-1 E* gene: residues corresponding to prM, envelope, penta-glycine and stretch of six histidine residues are shown in blue, red, orange and green, respectively. The two N-terminal residues (MV) are the result of placing the initiator codon in a Kozak consensus context. The arrows in panels A and B denote the site of prM signal peptide cleavage. (C) Western blot analysis of DENV-1 E antigen in soluble (S) and membrane (M) fractions of induced (I) and un-induced (UI) *P. pastoris* cells using EDIII-specific 24A12 mAb (1˚ antibody) in combination with anti-mouse IgG HRPO (2˚ antibody). Pre-stained protein markers were analyzed in lane M and their sizes (in kDa) are indicated on the left.

**Figure S2:** **Purification and characterization of recombinant DENV-1 E antigen** (A) Immobilized metal affinity (Ni-NTA) chromatographic purification of *P. pastoris*-expressed DENV-1 E antigen. Blue (solid) curve indicates UV absorbance at 280 nm and black (dashed) curve indicates imidazole step gradient. (B) Coomassie stained SDS-PAGE showing pool of eluted fractions in Lane E. (C) Western blot analysis of purified DENV-1 E protein (lane E) using EDIII-specific mAb 24A12 (1˚ antibody) in combination with anti-mouse IgG-HRPO (2˚ antibody). (D) Protein blot analysis of glycosylation of DENV-1 E protein using Con A-HRPO conjugate. Lane ‘E’ represents purified DENV-1 E protein. Lane ‘L’ represents ovalbumin as a positive glycoprotein control. Lane ‘T’ represents purified EDIII-T protein (EDIII domain of all four dengue serotype fused to each other) taken as negative controls. Lane ‘M’ represents pre-stained protein size markers. Marker sizes, in kDa, are shown to the left of panels B-D.

Figure S3: Sequence alignment of P. pastoris optimized DENV-1, 2, 3, 4 E showing similarities and differences in the amino acid sequences between four dengue serotypes. An asterisk (*) indicates positions which have a single, fully conserved residue and are highlighted in yellow background. Colon (:) indicates conservation between groups of strongly similar properties - scoring > 0.5 in the Gonnet PAM 250 matrix. Period (.) indicates conservation between groups of weakly similar properties - scoring =< 0.5 in the Gonnet PAM 250 matrix.
